# Contributions to the Pathology, Diagnosis, and Treatment of Angular Curvature of the Spine

**Published:** 1867-04

**Authors:** 


Contributions to the Pathology, Diagnosis, and Treatment of Angular Curvature of the Spine. By BENJAMIN LEE, M.D. Philadelphia: J. B. LIPPINCOTT & Co. 1867. pp. 129.

This little volume is neatly printed, but without an index or table of contents. It is made up of four interesting essays or articles, originally published in the *Medical Times*, of New York, and styled as follows:—1st, Initial Gastralgia. 2d, False Views. 3d, Correct Principles. 4th, Ten Type Cases. The author writes in an easy, interesting style; and the work will amply repay a careful perusal. For sale by S. C. GRIGGS & Co., 41 Lake St. Price $1.25.

